# The Electronic Properties of Cordycepin in the Adenine Nucleoside Landscape: A Theoretical Approach

**DOI:** 10.3390/molecules30112289

**Published:** 2025-05-23

**Authors:** Boleslaw T. Karwowski

**Affiliations:** Nucleic Acids Damage Laboratory, Faculty of Pharmacy, Medical University of Lodz, ul. Muszynskiego 1, 90-151 Lodz, Poland; boleslaw.karwowski@umed.lodz.pl

**Keywords:** Cordycepin (3′-deoxyadenosine), 7,8-dihydro-8-oxo-3′-deoxyadenosine, 8-hydroxy-3′-deoxyadenosie, ionisation potential, electron affinity, global reactivity descriptors, DFT

## Abstract

The anticancer activity of 3′-deoxyadenosine (Cordycepin, or dCor) is known to be linked to the inhibition of the MAPK/ERK signalling and Hedgehog pathways, as well as the termination of primer elongation by primase in DNA lagging-strand synthesis. In this study, the electronic properties of dCor, 7,8-dihydro-8-oxo-3′-deoxyadenosine (^OXO^dCor), and 8-hydroxy-3′deoxyadenosie (^HO^dCor), together with their spin densities, charge distributions, and global reactive descriptors, have been taken into consideration at the M06-2x/6-31++G** level of theory in the aqueous phase. It was found that dCor predominantly adopts a *3′-endo,anti* conformation, while ^OXO^dCor and ^HO^dCor adopt a *2′-endo,syn* conformation. Also, the keto form of oxidised dCor was found to be energetically preferred to its enolic form. The adiabatic ionisation potentials (AIPs) were noted as follows (in eV): 6.29 for dCor, 6.21 for ^OXO^dCor, and 6.17 for ^HO^dCor. The lowest adiabatic electron affinity among all the discussed adenine nucleosides analogues was assigned for ^OXO^dCor at 1.12 eV. A thorough analysis of the spin density distribution of the adiabatic radical cation reveals that it has a higher accumulation at N6 > C5 > C8 > 3 of dCor, C5 > N6 > N7 > O8 of ^OXO^dCor, and N6 > C5 > C8 > C2 of ^HO^dCor. The results suggest that Cordycepin is more easily converted to ^OXO^dCor and ^HO^dCor than canonical adenine nucleosides. Much like typical drugs, after its administration and release, Cordycepin is exposed to various physiological factors and can be exposed to ionisation radiation during combined therapy. These factors can influence the therapeutic potential of Cordycepin. Therefore, further studies on its stability are of utmost importance.

## 1. Introduction

The human body contains around 10^14^ cells, which are continuously exposed to harmful external and internal factors, including radiation, xenobiotics, and reactive oxygen and nitrogen species (ROS/RNS) [1]. The human genome, which consists of 3 × 10^9^ base pairs arranged in a precise sequence, regulates all cellular functions. Exposure to these unwanted factors can cause mutations, which may lead to carcinogenesis [2].

To date, more than 80 different types of DNA damage have been discovered and studied [3]. Fortunately, throughout evolution, cells have developed protective systems, including enzymatic antioxidants (such as catalases, peroxidases, and dismutases), low-molecular-weight particles such as uric acid and glutathione, and several DNA repair systems [4]. Among these, base excision repair (BER) is at the forefront of these defence systems [5]. Among all the canonical nucleobases, guanine (Gua) is the most susceptible to oxidation because of its low oxidation potential of 1.49 V. For adenine, thymine, and cytosine, their oxidation potentials are as follows (in V): 1.97, 2.11, and 2.14, respectively [6]. All of these nucleosides are exposed to ROS and RNS activity, which can lead to disruptions via the hydrogen atoms’ abstraction from or addition to the double bond of purine/pyrimidine moieties. Left unrepaired, these modifications can alter the pattern of nucleoside complementarity.

However, the generation of DNA damage in cancer cells is the “objective” of anticancer therapies, such as radiotherapy, chemotherapy, or combined treatments [7]. Unfortunately, little is known about the stability of nucleoside analogue-based chemotherapeutics in the presence of radiation, especially with regard to their electronic properties. Drug–radiation interactions have been investigated, but mainly in the context of tumour susceptibility [8]. Furthermore, with the increasing incidence of cancer, scientific attention has shifted to compounds used in traditional medicines.

**Figure 1:**
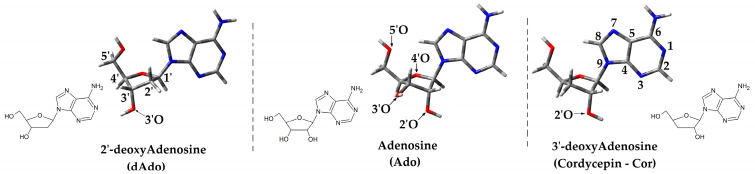
Graphical representation of adenine nucleoside (Ado, dAdo, Cor) structures calculated at the M062x/6-31++G** level of theory in the aqueous phase and atom numbering indications.

The 3′-deoxyadenosine Cordycepin (dCor) is a non-canonical nucleoside that was discovered in the fungus *Cordyceps sinensis* (Figure 1) [9]. It is a valuable compound that has long been used in traditional Chinese medicine [10]. Recently, the anticancer activity of Cordycepin has been discovered to be linked to the inhibition of the MAPK/ERK (mitogen-activated protein kinase/extracellular signal-regulated kinase) and Hedgehog pathways [11]. Furthermore, it has been linked to the absence of the 3′-hydroxyl group and the termination of primer elongation by primase in DNA lagging-strand synthesis [12].

Although the biological activity of Cordycepin has been well studied, its electronic properties have yet to be investigated. In view of the above, this article presents the first theoretical investigation into the influence of ionising radiation on the 3′-deoxy analogue of adenosine, i.e., Cordycepin [13].

## 2. Results

Assigning its electrical properties will go some way to improving the stability and reactivity of dCor in physiological fluids, as well as potentially reducing its susceptibility to oxidation during radiotherapy after oral or parenteral administration.

To this end, in this study, the structure of dCor was optimised at the M062x/6-31++G** level of theory in the aqueous phase and compared with canonical nucleosides, i.e., Ado (adenosine) and dAdo (2′-deoxyadenosine). The mentioned Minnesota functionals, discovered by Truhlar, which belong to hybrid meta exchange–correlation functionals with 54% Hartree–Fock exchange, have been used for geometry optimisation as well as the energies of all the discussed molecules [14]. Moreover, the self-consistent field methodology with tight convergence criteria has been used. Due to the significant influence of the first water solvation shell on geometry and electronic properties, Tomasi’s Conductor-like Polarised Continuum Model (CPCM) was chosen [15]. It should be noted that for the purpose of this study, applications with a dielectric constant ε = 78.4 cannot be directly considered for physiological fluids [16]. The complexities of the cytosol prohibited current quantum methodology or DFT (density functional theory) applications, limited by the current supercomputer power and efficiency. To match previous studies, the augmented polarised valence double-ζ basis set 6-31++G** has been chosen [17], as it is relatively cost-effective and efficient [18]. It should be pointed out that the systematic studies had similar results for geometry and energies in calculations performed by M06-2x and MP2 (Møller–Plesset perturbation theory) [19].

### 2.1. Spatial Geometry Analysis of 3′-Deoxyadenosine (Cordycepin)

All nucleosides, which contain sugar and nucleobase moieties, exhibit high flexibility, with more than forty thousand different conformers possible for each canonical nucleoside [15]. A thorough structural analysis revealed that Cordycepin preferably adopts a *3′-endo,anti*-conformation similar to that of Ado. In contrast, for dAdo*2′-endo,anti* was found to be energetically privileged (Table S1, Supplementary Materials).

**Figure 2:**
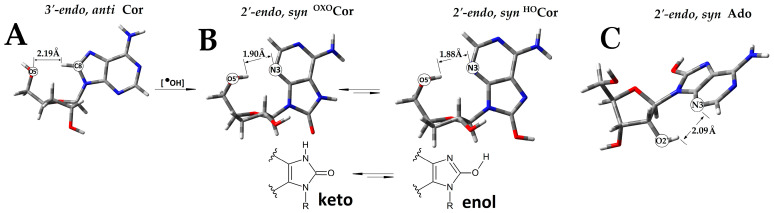
Graphical representation of the geometry of stable neutral conformers obtained at the M062x/6-31++G** level of theory in the aqueous phase: (**A**) 3′-deoxyadenosine (dCor), (**B**) their C8 oxidized analogues: ^OXO^dCor and ^HO^dCor with keto-enol tautomerism represented, and (**C**) Adenosine (Ado).

After oxygen attachment to the C8 and 7,8-dihydro-8-OXO-3′-deoxyAdenosine (^OXO^dCor) formation, a *2′-endo,syn* conformer was found to be the most stable. The same was noted for its enolic form, i.e., 8-hydroxy-3′-deoxyAdnosine (^HO^dCor) (Figure 2, Table S1). The canonical nucleoside *3′-endo,syn* conformation was found to be preferred for both enol and keto forms of 7,8-dihydro-8-OXO-2′-deoxyAdenosine (^OXO^dAdo), 7,8-dihydro-8-oxo-adenosine (^OXO^Ado), and 8-hydroxy-2′-deoxyAdenosine (^HO^dAdo), while for the 7,8-dihydro-8-oxo-adenosine (^HO^Ado)*2′-endo,* the *syn* conformer was noted as the most preferred (Table S1).

The oxidised nucleosides can exist in an equilibrium between enol and keto forms (Figure 2). The results obtained at the M062x/6-31++G** level of theory in the aqueous phase show that in each discussed case, the keto form is more stable than the enolic (Table S1). The following energetic differences of keto–enol pairs of most stabile conformers were assigned as follows (in kcal/mol): *2′-endo,syn*^OXO^dCor—*2′-endo,syn*^HO^dCor: 9.57; *3′-endo,syn* ^OXO^*dAdo-3′-endo,syn*^HO^dAdo: 9.78; and *3′-endo,syn*^OXO^Ado—*2′-endo,syn*^HO^Ado: 8.23.

Moreover, as shown in Figure 2A, the *syn* conformation of nucleosides permits hydrogen bond formation between the N3 and HO5′ group, increasing its stability. For the discussed derivatives ^OXO^dCor and ^HO^dCor, the distance was 1.9 Å and 1.88 Å, respectively, while for ^OXO^Ado, ^HO^Ado, ^HO^dAdo, and ^OXO^dAdo, the distance increased up to (in Å) 3.88, 5.22, 3.87, and 3.87, respectively. However, for ^HO^Ado, an additional HO2′-N3 interaction could be observed (2.0 Å) (Figure 2B).

The assigned difference between the *syn* and *anti*-conformers of ^OXO^dCor and ^HO^dCor was ~6.95 kcal/mol, while for canonical 8-OXO or 8-HydrOxy adenine nucleosides, it was found that the difference ranged between 0.04 and 2.10 kcal/mol (Table S1).

To simplify the notation for further consideration, only the following conformers are discussed and referred to, as they are the most stable: dCor, ^OXO^dCor, ^HO^dCor, Ado, ^OXO^Ado, ^HO^Ado and dAdo, ^OXO^dAdo, and ^HO^dAdo.

### 2.2. The Charge and Spin Distribution Analysis of 3′-Deoxyadenosine and Adenine Nucleoside Radicals

The spin density and charge distribution were calculated at the M062x/6-31++G** level of theory in the condensed phase (CPCM model) using Hirshfeld’s methodology [20]. Non-equilibrated and equilibrated solvent–solute interactions were taken into consideration. As expected for dCor, ^OXO^dCor, and ^HO^dCor, the spin density and charge distribution underwent reorganisation during solvent–solute interaction mode changes (Table S2). After electron loss by dCor, ^OXO^dCor, and ^HO^dCor (one-electron oxidisation) and the formation of a non-equilibrated vertical cation state, the unpaired electron (spin) settled almost exclusively (~100%) on the purine moiety in all the cases under discussion. The positive charge was distributed between the sugar (~30%) and purine (~70%) moieties, as shown in Table S2.

**Figure 3:**
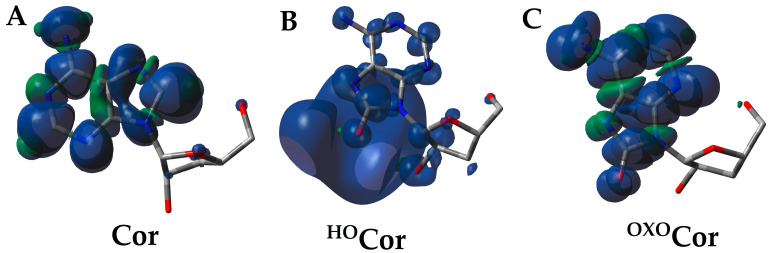
Visualisation of the spin distribution of (**A**) dCor, (**B**) ^HO^dCor, and (**C**) ^OXO^dCor in their non-equilibrated vertical anion states, calculated at the M062x/6-31++G** level of theory in the aqueous phase.

The adoption by the molecule of an extra electron causes the formation of a radical anion. For parental 3′-deoxyadenosine, the spin was always located on the adenine, together with a negative charge. In the cases of ^OXO^dCor and ^HO^dCor, in the non-equilibrated vertical anion radical state, the unpaired electron was dispersed over the sugar and base moieties at a ratio of 2:98% in the case of ^OXO^dCor and 24:76% for ^HO^dCor (Figure 3). Surprisingly, a negative charge was noted at a rate of 97% and 92% on the purine subunit in both cases (Table S2). After solvent and solute relaxation, the equilibrated vertical and adiabatic radical anions were formed with the charge and spin settling on the heterocyclic compound (over 96%), which is similar to the results obtained for canonical nucleosides and their derivatives (Table S2).

**Figure 4:**
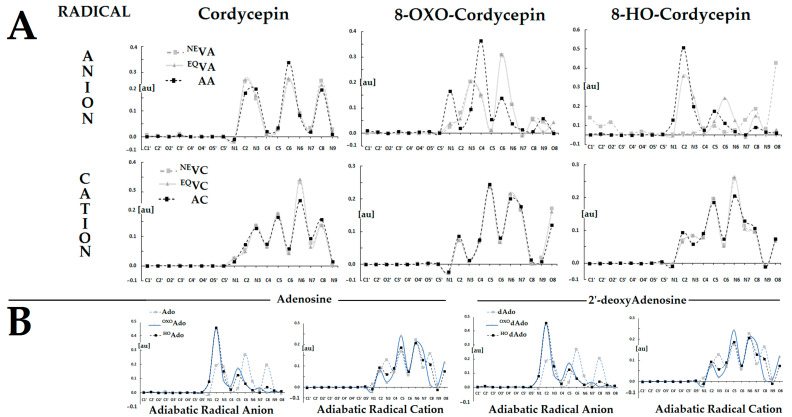
(**A**) Spin distribution within dCor, ^OXO^dCor, and ^HO^dCor calculated at the M062x/6-31++G** level of theory in the condensed phase. The raw data of the charge and spin distribution are given in Table S3. (**B**) Spin distribution within adiabatic radical anion and cation states of canonical nucleosides and their oxidised analogues: Ado, ^OXO^Ado, ^HO^Ado and dAdo, ^OXO^dAdo, and ^HO^dAdo, obtained at the M062x/6-31++G** level of theory in the aqueous phase. The raw data are given in Table S3.

A thorough analysis of the non-equilibrated/equilibrated vertical and adiabatic cations formed by dCor, ^OXO^dCor, and ^HO^dCor reveals the same pattern of spin accumulation. As shown in Table S2, for the series of dCor, the spin accumulation was predominantly found at the N6 > C5 > N3 > C8 atoms, with ^OXO^dCor at C5 > N6 > N7 > O8 and ^HO^dCor at N7 > C5 > N7 > C8. In all of the above cases, C5 was the most susceptible carbon atom to the further addition of radicals (e.g., ●OH). Similar results were noted for the adiabatic cation state of parental nucleosides (Figure 4).

The situation becomes different after the adoption of an extra electron. In the case of Cordycepin, the extra electron settled at the C6 > C2 > C8 carbons. The same was noted for the Ado and dAdo adiabatic radical anion. The conversion of dCor to ^OXO^dCor and its enolic form ^HO^dCor was found to involve the adoption of an extra electron in the initial state (^NE^VA) by C6 and O8 atoms, respectively. After solvent equilibration, a higher spin density was found at C6, C4, and N3 of ^OXO^dCor, while for ^HO^dCor, it was distributed over the C2, C6, and C8 atoms. After spatial geometry relaxation and the achievement of the adiabatic anion state, the unpaired electron was located predominantly at the C4 and C2 carbon atoms of ^OXO^dCor and ^HO^dCor, respectively (Figure 3). It should be pointed out that in the adiabatic radical anion states of ^OXO^dA, ^HO^dA and ^OXO^Ado, and ^HO^Ado, a higher spin density was observed at C2 atom of the adenine moiety.

### 2.3. Ionisation Potentials (IPs) of 3′-Deoxyadenosine and a Comparison with Adenine Nucleosides

One of the parameters determining molecule stability and susceptibility to one-electron oxidation or reduction is a molecule’s tendency to electron loss or adoption, which is referred to as either its ionisation potential or electron affinity. The loss of an electron by neutral molecules generates a radical cation state, while the gain of an additional electron gives rise to radical anion formation [21].

The above processes in the initial step occur without solvent–solute reorganisation (in a non-equilibrated state) and determine the initial antioxidant properties. Therefore, the vertical ionisation potential (VIP) or electron affinity (EA) in non-equilibrated solvent–solute interaction (NE) modes should be taken into consideration [22].

As was expected, dCor exhibited a higher ^NE^VIP than ^OXO^dCor and ^HO^dCor as follows: Cor > ^OXO^Cor > ^HO^Cor (Table 1). The equilibration of the solvent–solute interaction (EQ) in which only the solvation shell is relaxed causes a decrease in VIP of ~1.0 eV. Furthermore, the following order of ^EQ^VIP was found: dCor > ^OXO^dCor > ^HO^dCor (Table 1).

Molecular geometry relaxation gives rise to an adiabatic state. The following adiabatic ionisation potential (AIP) arrangement was observed for 3′-deoxyadenosine and its analogues: dCor > ^OXO^dCor > ^HO^dCor. Subsequently, the AIP decreases to as little as 5.93 eV for *3′-endo,syn*^HO^dAdo, which is the lowest value of all the discussed nucleosides (Table 1). For 2′-deoxyguanosine, at the same level of theory, the assigned AIP approached 5.83 eV (the average value of canonical forms in dsDNA) [23].

### 2.4. Electron Affinity (EA) of 3′-Deoxyadenosine and a Comparsion with Adenine Nucleosides

The ability of molecules to adopt an additional electron with subsequent radical anion formation is represented by the electron affinity (EA) parameters, as presented in Table 1. The obtained results show that the electron attachment process was noted as unfavourable at the initial point (in a non-equilibrated solvent–solute interaction state); please see Table 1.

The 3′-deoxyadenosine derivatives adopted negative values for the non-equilibrated vertical electron affinity (VEA^NE^). The ^NE^VEA values for the dCor and ^OXO^dCor were close to zero, while for ^HO^dCor, it was found to be significantly lower, i.e., −0.59 eV.

The adoption of an additional electron by the nucleoside and subsequent solvent relaxation lead to equilibrated vertical radical anion formation. The following order of equilibrated vertical electron affinity (VEA^EQ^) was noted: dCor ≈ ^OXO^dCor > ^HO^dCor. Further geometry relaxation caused the formation of an adiabatic radical anion. The adiabatic electron affinity (AEA) values were as follows: ^HO^dCor > dCor > ^OXO^dCor. For both canonical nucleosides, i.e., dAdo and Ado, and their derivatives, the order of AEA values was as follows: ^OXO^dAdo > dAdo > ^HO^dAdo.

Among all the discussed molecules, 7,8-dihydro-8-oxo-adenosine exhibited the highest adiabatic electron affinity of 1.49 eV, while 7,8-dihydro-8-oxo-3′-deoxyadenosine had the lowest, measured at 1.12 eV.

### 2.5. Global Reactivity Descriptors of 3′-Deoxyadenosine and a Comparsion with Canonical Adenine Nucleosides

The energy gap between the highest and lowest occupied molecular orbitals (HOMO, LUMO, respectively) denoted as Δ*E*^H-L^ = *E^HOMO^* − *E^LUMO^* is a convenient descriptor of molecular stability and chemical reactivity [24,25]. A greater Δ*E*^H-L^ value indicates higher molecular stability. The 8-hydroxy derivatives of dCor, dAdo, and Ado exhibited lower Δ*E*^H-L^ values, thus predicting the highest chemical reactivity among all the discussed molecules (Table 2). The lowest value was calculated for ^HO^Ado (7.35 eV). In contrast, a higher Δ*E*^H-L^ value was noted for the unmodified nucleosides in the following order: dAdo > Ado > Cor. The subsequent conversion to the 7,8-dihdro-8-oxo analogue reduced the Δ*E*^H-L^ value (in eV) by 0.15, 0.13, and 0.06 for dAdo, Ado, and Cor, respectively.

Based on Koopmans’ theorem for closed-shell molecules, the following parameters can be calculated (in eV): chemical hardness (η = 0.5(LUMO-HOMO)) and softness (*S* = 1/η), the global electrophilicity index (ω = μ^2^/2η), and electronic chemical potential (*μ* = 0.5(LUMO + HOMO)). The parameters were also calculated for the adiabatic molecular states. It should be pointed out that the orbital energies can be obtained by the Kohn–Sham calculation with different long-range corrected functionals [26]. Using the above leads to the satisfactory results of Koopmans’ theorem [27]. The above is coincident with Janak’s theorem since the orbital energies are related to the occupation number (the energy difference between the cation/anion and neutral state corresponds to the IP and EA, respectively) [28,29].

Among all the molecules under discussion, the unmodified nucleoside exhibited the highest η value in the following order according to Koopmans’ theorem and the adiabatic state mode: dAdo > Ado > Cor and Ado > dAdo > Cor, all of which indicates their stability. The lowest values were found for ^HO^dAdo (3.61 eV) and ^OXO^dCor (3.67 eV) and can be perceived as more reactive than the others, after achieving an adiabatic state. Based on the above results, it can be concluded that the enolic tautomer becomes more reactive than the keto tautomer in all cases (Table 2).

According to Pérez, “*molecules can be regarded as strong electrophiles when the global electrophilicity index (ω) is above* 1.5 *eV, weak when below* 0.8 *eV, and moderate electrophiles for ω values between* 1.5 *and* 0.8 *eV*” [30].

The results presented in these studies show that, according to Koopmans’ theorem, all the discussed molecules are strong electrophiles in their neutral state (Table 2). After electron loss/adoption and related adiabatic radical anion or cation formation, the resulting species become weaker electrophiles with ω values below 0.8 eV, except for ^OXO^dCor, ^HO^dAdo, and ^HO^Ado, which exhibit moderate electrophilicity: 0.88, 0.81., and 0.91, respectively. ^HO^Ado was found to be the weakest electrophile, with a global electrophilicity index of 0.68 eV.

The electronic chemical potential (μ) indicates the tendency of the investigated system to electron loss or adoption. A high negative μ value suggests that the molecules are good electron acceptors. Conversely, a lower negative μ value suggests a predisposition for electron donation.

As expected, the electronic chemical potential decreases after adiabatic cation/anion formation in comparison to the corresponding neutral state.

## 3. Discussion

Reactive oxygen species (ROS) and reactive nitrogen species (NOS) are found in abundance within the internal and external cellular environments and can appear as products of the activity of various factors, including ionisation radiation, xenobiotics, and photosynthesis, and as the incomplete four-electron reduction of oxygen in the oxygen deactivation process, i.e., O_2_ → O_2_^•−^ → H_2_O_2_ → •OH + OH^−^ → 2H_2_O [31]. ROS and NOS are important cell messengers that can play a significant role in processes such as control, inflammation, proliferation, and cell death signal induction, depending on their source, concentration, duration, and compartmentalisation. Their presence in physiological fluids is generally rather brief: (•OH) 10^−10^ s; (O_2_^•−^) 10^−6^ s; (ROOH, H_2_O_2_) stable; (^1^O_2_) 10^−6^ s; (HOCl and HOBr) a few minutes; and (ONOO^−^ peroxynitrite) 10^−3^ s [32]. The hydroxyl radical is noticeably the most reactive, with a constant reaction rate of 10^9^–10^10^ M^−1^s^−1^ and redox potential *E*_0_ (H_2_O/•OH) = 2.32 V at pH = 7 [33]. It should be pointed out that because of the diffusion rate constant, •OH cannot be deactivated enzymatically [34,35]. Therefore, these reactive molecules are harmful species that can alter amino acids and the nucleoside/nucleotide structure by proton abstraction from or addition to unsaturated bonds.

Furthermore, molecules administered from other sources such as anticancer/antiviral drugs, after administration, liberation, and distribution, are exposed to the same harmful factors as cellular molecules. Many therapeutic compounds are built on the nucleoside’s leading structure or are part of therapeutic nucleic acids [36]. The rapid advances in different genetic therapies using oligonucleotides—native or modified (phosphorothioates)—raises questions about their stability. Most of the answers have been given in the context of enzymes, e.g., the stability of DNA/RNA analogues in the serum [37]. However, little is known about the stability molecules in the presence of ROS and NOS in cytosol or extracellular fluids. This is particularly crucial in the case of combined therapy in which ionisation radiation is used, which results in increased concentrations of ROS and NOS.

Therefore, in this study, the electronic properties of 3′-deoxyadenosine (Cordycepin—Cor) were taken into theoretical consideration. Used in traditional Chinese medicine, Cordycepin is a promising analogue of 2′-deoxyadenosine found in *Cordyceps sinensis* [38].

After cell administration, dCor becomes part of the intracellular pool of nucleosides, which are abundant in cytosol before they are incorporated into DNA or RNA, as well as being converted to ATP, cAMP, etc.: ATP: 3152, GTP: 468, UTP: 567, CTP: 278; and for 2′-deoxynucleotides: dATP: 24, dGTP: 5.2, dCTP: 29, and dTTP: 37 [39] (all concentrations are given in μM) [39].

The lowest ionisation potential (in eV) of all native nucleobases and nucleosides has been denoted for guanine and 2′-deoxyguanosine, i.e., 8.1 eV [40] and 8.6 eV [41], respectively (in the gaseous phase). Ade and dAdo have been measured at 8.26 and 8.9 eV, respectively [42].

In this study, Cordycepin’s ionisation potential: ^NE^VIP, ^EQ^VIP and AIP, calculated at the M062x/6-31++G** level of theory aqueous phase, was found to be as follows (in eV): 7.63, 6.56, and 6.29. For the native Ado and dAdo, the AIPs were noted as higher (Table 1), indicating that Cor is more prone to radical cation formation and, therefore, can be predicted to be less stable than the canonical nucleoside under ionisation radiation conditions.

The situation changes after the formation by adenine nucleoside analogues of 8-OXO derivatives. The following order of AIP was obtained: ^OXO^Cor > ^OXO^Ado > ^OXO^dAdo. Surprisingly, for the enolic form, the order of this parameter was different, i.e., ^HO^Cor > ^HO^Ado > ^HO^dAdo. It should be borne in mind that for all the discussed C8 oxidised molecules which exist in the keto-enol tautomer equilibrium, the keto form is energetically privileged (Table S1).

On the other hand, the adoption of an additional electron led to the formation of ^NE^VEA, ^EQ^VEA, and AEA, which describes molecular electron affinity. Cordycepin exhibited the following values for the above parameters: −0.01, 1.04, and 1.38, respectively (Table 1). For ^OXO^dCor, both VEA values were at the same level as their native counterparts, while the AEA value decreased to 1.12 eV. For the enolic form, the ^NE^VEA value was measured at the significantly lower value of −0.59; however, AEA was at a similar level to dCor. These results indicate that ^HO^dCor is more resistant to one-electron reduction at the initial point of negative charge attachment (e.g., with a solvated electron).

A comparison of 3′-deoxyadenosine AEA with canonical nucleosides revealed the following order: ^OXO^Ado > ^OXO^dAdo > ^OXO^dCor and ^HO^dCor > ^HO^Ado > ^HO^dAdo. Among all of the oxidised derivatives, ^OXO^Ado had the highest value at 1.49 eV, while ^HO^dAdo had the lowest (1.21 eV). The above indicates that not only does the modification in the nucleobase structure influence the electronic parameters of the nucleoside, but it also causes changes in the sugar moiety.

The charge distribution within the discussed nucleosides was observed between the sugar and nucleobase moieties (Table S2), with an unpaired electron (spin) mainly located on the adenine subunit. It should be noted that the positive charge on the sugar moiety was more pronounced (23–38%) compared to that found in the anions. In contrast, the spin was almost entirely localised (close to 100%) on the base moieties at both the vertical and adiabatic stages, except for ^HO^dCor, for which 24% of the spin was observed on 3-deoxyribose (Figure 3).

Similar findings were noted for the adiabatic radical cation and anion of canonical nucleosides, regardless of the presence of an additional oxygen atom in their structures (Table S2). This result indicates some similarity between Cordycepin and adenosine/2′-deoxyadenosine; however, a careful analysis of the spin distribution revealed differences (Figure 3).

First of all, the unpaired electron settled on the following atoms of dCor: N6 (28%), C5 (17%), and C8~N3 (14%), with only minor differences observed between non-equilibrated, equilibrated, and adiabatic states. Similar results were found for ^OXO^dCor: C5 (24%), N6 (20%), N7 (17%), and O8 (16%), while for ^HO^dCor, the following order was assigned: N6 (21%), C5 (19%), N7 (13%), and C8 (11%). For adiabatic radical cation states, the spin was localised on the following atoms: N6 (22%), C5 (17%), C8 (12%), and N3 (13%) of both Ado and dAdo. For the keto forms of oxidised nucleosides (^OXO^dAdo and ^OXO^Ado), the distribution was as follows: C5 (24%), N6 (21%), N7 (18%), and O8 (12%), while for enolic derivatives, it was as follows: N6 (21%), C5 (19%), N7 (13%), and C8 (11%). These results suggest that further interactions of the molecules with hydroxyl radicals may lead to the formation of similar molecular analogues due to the similarity in spin density distribution.

The appearance of the additional electron in the molecules causes the formation of radical anions. As shown in Figure 3A, the spin distribution changes in line with the system relaxation: non-equilibrated → equilibrated solvent–solute interaction → adiabatic. After structure relaxation, the additional electron was primarily found at a rate of 46% on C2 of ^HO^dCor, 36% on C4 of ^OXO^dCor, and on C6 (29%), N3 (19%), C8 (18%), and C2 (17%) of dCor, indicating the driving force of O8.

In the case of canonical nucleosides dAdo and Ado, the negative charge was localised as follows: C6 (27%), C8 (21%), C2 (19%), and N3 (18%). The situation appears to be simpler after the oxygen’s attachment to C8. The spin density was mainly accumulated at a rate of ~45% on C2 of ^HO^Ado, ^HO^dAdo, ^OXO^Ado, and ^OXO^dAdo. All the above results indicate the differences between 3′-deoxyadenosine, adenosine, and 2′-deoxyadenosine derivatives after one-electron reduction. The C4 atom of ^OXO^dCor was the most prone to ●OH addition, while for the other oxidised molecules, the C2 atom displayed the highest susceptibility. In the case of the unmodified nucleoside, the highest predisposition for ●OH addition was observed on the C6 atom in each anion radical case.

While differences in spin distribution among the discussed molecules were observed, the results of global reactivity descriptors shed light on the hardness, softness, electrophilicity, and electronic chemical potential of molecules [43]. A comparison of these parameters calculated according to Koopmans’ theorem [24,25,44] and the adiabatic mode (presented in Table 2) elucidated the highest hardness in the case of dCor, dAdo, and Ado. The above are in good agreement with the assigned ionisation potential, which decreased after the oxygen’s attachment to C8 (Table 1). Moreover, the enolic forms become more reactive than keto in all cases.

## 4. Materials and Methods

The spatial structures of the investigated short oligonucleotides were constructed using GaussView 5 software [45] and are depicted in Figure 1. For all calculations, the density functional theory (DFT) has been used as follows: M62x/6-31++G** in the condensed phase. The applied Pople basis set contains 5s4p3d2f and 4s3p2d functions for heavy atoms and hydrogen, respectively [46]. All experiments were carried out in the aqueous phase using the polarised conductor-like continuum model (CPCM) with a water dielectric constant ε = 78.4 [47]. The spin and charge distribution were achieved and analysed using Hirshfeld’s methodology [48] at the mentioned level of theory. The solvent effect was investigated in two modes of C-PCM, i.e., at non-equilibrium (NE) and equilibrated (EQ) [22,49].

The following energy notation was used: the *E*_geometry_^charge^ of the molecule (e.g.,: geometry of neutral ground state *E*_0_, charge of neutral molecules *E*^0^) is described as *E*_0_^0^; the vertical cation/anion in the non-equilibrated solvent–solute interaction mode is described as *E*_0_^+(NE)^/*E*_0_^−(NE)^; the vertical cation/anion in the equilibrated solvent–solute interaction mode is described as *E*_0_^+(EQ)^/*E*_0_^−(EQ)^; and the adiabatic cation/anion is described as *E*_+_^+^/^+^*E*_−_^−^ [42].

The differences (eV) between the electronic states were represented as outlined: VIP^NE^ = *E*_0_^+(NE)^ − *E*_0_^0^ (vertical ionisation potential in the NE mode); VIP^EQ^ = *E*_0_^+(EQ)^ − *E*_0_^0^ (vertical ionisation potential in the EQ mode); AIP = *E_+_*^+^ − *E*_0_^0^ (adiabatic ionisation potential in the relaxed solvent mode); VEA^NE^ = *E*_0_^−(NE)^ − *E*_0_^0^ (vertical electron affinity in the NE mode); VEA^EQ^ = *E*_0_^−(EQ)^ − *E*_0_^0^ (vertical electron affinity in the EQ state); and AEA = *E*_0_^0^ − *E*_−_^−^ (adiabatic electron affinity) [42].

All of the theoretical experiments were performed using the Gaussian G16 (version C.01) software package [50].

## 5. Conclusions

In this study, the electronic properties together with the spin density distribution and global reactive descriptors of 3′-deoxyadenosine (dCor), 7,8-dihydro-8-oxo-3′-deoxyadenosine (^OXO^dCor), and 8-hydroxy-3′deoxyadenosie (^HO^dCor) were taken into consideration and compared with canonical adenine nucleosides. The results of the presented studies were discussed at the M06-2x/6-31++G** level of theory in the aqueous phase in all cases.

Among all conformers of Cordycepin and Ado, *3′-endo,anti* has been found to be the most stable one, while the *2′-endo,syn* conformation was assigned as preferred in the case of ^OXO^dCor, ^HO^dCor, and ^HO^Ado.

The keto form of C8 oxidising 3′-deoxyadenosnie, i.e., ^OXO^dCor, was energetically preferred to its enolic counterpart (^HO^dCor), which is similar to the results obtained for canonical adenosine nucleosides, i.e., ^OXO^dA > ^HO^dAdo and ^OXO^Ado > ^HO^Ado.

The vertical and adiabatic ionisation potentials decrease as follows: dCor > ^OXO^dCor > ^HO^dCor. The AIPs were found as follows (in eV): dCor (6.29), ^OXO^dCor (6.21), and ^HO^dCor (6.17 eV). They were measured higher than those for Ado and dAdo after oxidation.

The lowest adiabatic affinity among all the discussed adenine nucleosides’ analogues was found for ^OXO^dCor at 1.12 eV.

A thorough analysis of the spin density distribution of the adiabatic radical cation revealed a higher accumulation at N6 > C5 > C8 > 3 of dCor, C5 > N6 > N7 > O8 of ^OXO^dCor, and N6 > C5 > C8 > C2 of ^HO^dCor. For the adiabatic radical anion, the spin density distribution was as follows: C6 > C8 > N3 > C2 of dCor and mainly distributed on C2 of ^OXO^dCor and ^HO^dCor.

The global reactivity descriptors, which were calculated according to Koopmans’ theorem in the adiabatic state mode, exhibited the greatest hardness in the cases of dCor, dAdo, and Ado.

According to Pérez’s global electrophilicity index (ω), all the discussed nucleosides can be predicted as strong electrophiles according to Koopmans’ theorem. After related adiabatic radical anion/cation formation, they become weaker electrophiles with ω values below 0.8 eV, except for ^OXO^dCor, ^HO^dAdo, and ^HO^Ado, which showed moderate electrophilicity at 0.88, 0.81, and 0.91, respectively. ^HO^Ado was found to be the weakest electrophile, with a global electrophilicity index of 0.68 eV.

A comparative analysis of the obtained data indicates that Cordycepin can be converted to ^OXO^dCor and ^HO^dCor more easily than canonical adenine nucleosides. Therefore, its therapeutic potential in combined anticancer therapies could change and warrants further investigation.

## Figures and Tables

**Table 1:** The electronic properties calculated at the M062x/6-31++G** level of theory in the aqueous phase of dCor, ^OXO^dCor, and ^HO^dCor and adenine nucleoside derivatives (in eV) denoted as follows: vertical ionisation potential (VIP) and vertical electron affinity (VEA), considered in non-equilibrated (NE) and equilibrated (EQ) solvent–solute interaction modes, adiabatic ionisation potential (AIP), and adiabatic electron affinity (AEA). The raw data of the orbitals are given in the Supplementary Materials (Table S5).

AIP	^EQ^VIP	^NE^VIP	^NE^VEA	^EQ^VEA	AEA	Canonical Nucleosides					
**dCor**						AIP	AEA	AIP	AEA	AIP	AEA
6.29	6.56	7.63	−0.01	1.04	1.38	**dAdo**		**^OXO^dAdo**		**^HO^dAdo**	
**^OXO^dCor**						6.31	1.38	6.12	1.39	5.93	1.21
6.21	6.53	7.58	−0.01	1.04	1.12	**Ado**		**^OXO^Ado**		**^HO^Ado**	
**^HO^dCor**						6.31	1.39	6.16	1.42	6.18	1.34
6.17	6.50	7.55	−0.59	0.96	1.39						

**Table 2:** The reactivity descriptors obtained at the M062x/6-31++G** level of theory in the aqueous phase of 3′-deoxyadenosine and adenine nucleoside derivatives, i.e., canonical and oxidised. The energies of valence molecular orbitals (HOMO, LUMO) and their energy gaps (Δ*E^H-L^*) are given in eV. The parameters calculated after radical anion/cation spatial structure relaxation (adiabatic mode) and compared with those obtained according to Koopmans’ theorem (Table S4).

Comp.	Energy (eV)			Theory/Mode	Parameters (eV)			
	HOMO	LUMO	|*ΔE^H-L^*|		*η*	*S*	*μ*	*ω*
dCor	−7.64	−0.10	7.54	Koopmans/ Adiabatic	3.77/3.83	0.27/0.26	−3.87/−2.46	1.98/0.79
^OXO^dCor	−7.62	−0.14	7.48		3.74/3.67	0.27/0.27	−3.88/−2.54	2.02/0.88
^HO^dCor	−7.58	−0.14	7.44		3.72/3.78	0.27/0.26	−3.86/−2.39	2.00/0.76
dAdo	−7.66	−0.08	7.57	Koopmans/Adiabatic	3.79/3.84	0.26/0.26	−3.87/−2.47	1.98/0.79
^OXO^dAdo	−7.55	−0.13	7.42		3.71/3.76	0.27/0.27	−3.84/−2.37	1.99/0.75
^HO^dAdo	−7.50	−0.13	7.37		3.68/3.64	0.27/0.27	−3.81/−2.43	1.97/0.81
Ado	−7.67	−0.13	7.54	Koopmans/Adiabatic	3.77/3.87	0.27/0.26	−3.90/−2.47	2.02/0.79
^OXO^Ado	−7.58	−0.17	7.41		3.71/3.84	0.27/0.26	−3.88/−2.29	2.03/0.68
^HO^Ado	−7.52	−0.17	7.35		3.68/3.61	0.27/0.28	−3.85/−2.56	2.01/0.91

## Data Availability

Data are contained within the article and Supplementary Materials.

## Supplementary Materials

The following supporting information can be downloaded at: https://www.mdpi.com/article/10.3390/molecules30112289/s1, Table S1. The calculated energy in Hartree on M06-2x/6-31++G** level of theory in aqueous phase of broder conformers of 3′-deoxyadenosine (dCor), 7,8-dihydro-8-OXO-3′-deoxyadenosine (^OXO^dCor), 8-hydroxy-3′-deoxyadenosine (^HO^dCor), 2′-deoxyadenosine (dAdo), 7,8-dihydro-8-OXO-2′-deoxyadenosine (^OXO^dAdo), 8-hydroxy-2′-deoxyadenosine (^HO^dAdo), Adenosine (Ado), 7,8-dihydro-8-OXO-adenosine (^OXO^Ado), 8-hydroxy-adenosine (^HO^Ado). Table S2. Hirshfeld charge (Q) and spin (S) distribution calculated at the M06-2x/6-31++G** level of theory in the aqueous phase. Vertical Cation (^NE^VC) (NE-non-equilibrated), Vertical Cation (^EQ^VC) (EQ-equilibrated), Adiabatic Cation (AC) and Vertical Anion (^NE^VA) (NE-non-equilibrated), Vertical Anion (^EQ^VA) (EQ-equilibrated), Adiabatic Anion (AA). Ri: ribose, 2-dR: 2-deoxyribose, 3-R: 3-deoxyribose, Ade: adenine. Table S3. The raw data of the charge and spin distribution within dCor, ^OXO^dCor, ^HO^dCor and adiabatic radical anion/cation states of canonical nucleosides and their oxidised analogues: Ado, ^OXO^Ado, ^HO^Ado, dAdo, ^OXO^dAdo, ^HO^dAdo calculated at the M062x/6-31++G** level of theory in the aqueous phase. Hirshfeld charge (Q) and spin (S) distribution calculated at the M06-2x/6-31++G** level of theory in the aqueous phase. Vertical Cation (^NE^VC) (NE-non-equilibrated), Vertical Cation (^EQ^VC) (EQ-equilibrated), Adiabatic Cation (AC) and Vertical Anion (NEVA) (NE-non-equilibrated), Vertical Anion (^EQ^VA) (EQ-equilibrated), Adiabatic Anion (AA). Table S4. The energies in Hartree calculated at the M06-2x/6-31++G** level of theory in the aqueous phase. Vertical Cation (^NE^VC) (NE-non-equilibrated), Vertical Cation (^EQ^VC) (EQ-equilibrated), Adiabatic Cation (AC) and Vertical Anion (^NE^VA) (NE-non-equilibrated), Vertical Anion (^EQ^VA) (EQ-equilibrated), Adiabatic Anion (AA). Ri: ribose, 2-dR: 2-deoxyribose, 3-R: 3-deoxyribose, Ade: adenine.
