# Supplementary material for: The Electronic Properties of Cordycepin in the Adenine Nucleoside Landscape: A Theoretical Approach
Source: Molecules. 2025 May 23;30(11):2289. doi: 10.3390/molecules30112289 (PMC12155987; doi:10.3390/molecules30112289)

The electronic properties of Cordycepin on the adenine nucleoside landscape: a theoretical approach

Boleslaw T. Karwowski

Nucleic Acids Damage Laboratory, Faculty of Pharmacy, Medical University of Lodz, ul. Muszynskiego 1, 90-151 Lodz, Poland; Boleslaw.Karwowski@umed.lodz.pl

Table S1.

The calculated energy in Hartree on M06-2x/6-31++G** level of theory in aqueous phase of broder conformers of 3′-deoxyadenosine (dCor), 7,8-dihydro-8-OXO-3′-deoxyadenosine (^{OXO}dCor), 8-hydroxy-3′-deoxyadenosine (^{HO}dCor), 2′-deoxyadenosine (dAdo), 7,8-dihydro-8-OXO-2′-deoxyadenosine (^{OXO}dAdo), 8-hydroxy-2′-deoxyadenosine (^{HO}dAdo), Adenosine (Ado), 7,8-dihydro-8-OXO-adenosine (^{OXO}Ado), 8-hydroxy-adenosine (^{HO}Ado)

	**dCor**		**^{OXO}dCor**		**^{HO}dCor**	
	2′-endo (*S*)	*3′-endo* (*N*)	*2′-endo* (*S*)	*3′-endo*(*N*)	*2′-endo* (*S*)	*3′-endo* (*N*)
syn	-888.048916	-888.050178	-963.286365	-963.277226	-963.271113	-963.261529
anti	-888.052561	-888.054851	-963.275283	-963.277173	-963.260017	-963.260001
	**dAdo**		**^{OXO}dAdo**		**^{HO}dAdo**	
syn	-888.049594	-888.050048	-963.276434	-963.277132	-963.261418	-963.261555
anti	-888.054061	-888.052805	-963.276076	-963.276967	-963.261003	-963.261101
	**Ado**		**^{OXO}Ado**		**^{HO}Ado**	
syn	-963.246196	-963.246637	-1038.472326	-1038.473913	-1038.460801	-1038.458066
anti	-963.249693	-963.250259	-1038.473556	-1038.473848	-1038.457448	-1038.457817

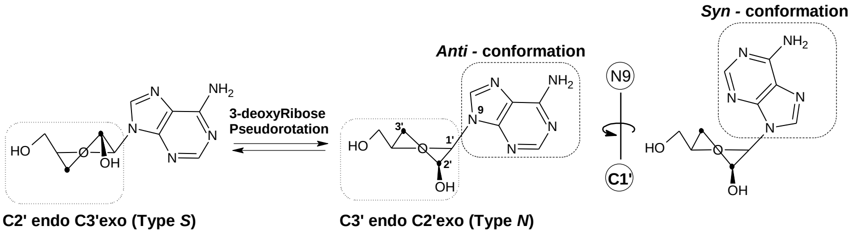

Table S2

Hirshfeld charge (**Q**) and spin (**S**) distribution calculated at the M06-2x/6-31++G** level of theory in the aqueous phase . Vertical Cation (^{NE}**VC**) (NE-non-equilibrated), Vertical Cation (^{EQ}**VC**) (EQ-equilibrated), Adiabatic Cation (**AC**) and Vertical Anion (^{NE}**VA**) (NE-non-equilibrated), Vertical Anion (^{EQ}**VA**) (EQ-equilibrated), Adiabatic Anion (**AA**). **Ri**: ribose, **2-dR**: 2-deoxyribose, **3-R**: 3-deoxyribose, **Ade**: adenine.

	**Neutral**	^{NE}**VA**		^{EQ}**VA**		**AA**		^{NE}**VC**		^{EQ}**VC**		**AC**	
	Q	*S*	*Q*	*S*	*Q*	*S*	*Q*	*S*	*Q*	*S*	*Q*	*S*	*Q*
	**dCor**												
**3-dR**	0.28	0.03	0.16	0.02	0.20	0.01	0.19	0.00	0.38	0.00	0.35	0.00	0.38
**Ade**	-0.28	0.97	-1.16	0.98	-1.20	0.99	-1.19	1.00	0.62	1.00	0.65	1.00	0.62
	^{OXO}**dCor**												
**3-dR**	0.16	0.02	0.03	0.01	0.09	0.04	0.06	0.00	0.26	0.00	0.23	0.01	0.31
**Ade**	-0.16	0.98	0.97	0.99	-1.09	0.96	-1.06	1.00	0.74	1.00	0.77	0.99	0.69
	^{HO}**dCor**												
**3-dR**	0.18	0.24	-0.08	0.01	0.11	0.02	0.10	0.00	0.27	0.00	0.24	0.00	0.32
**Ade**	-0.18	0.76	-0.92	0.99	-1.11	0.98	-1.10	1.00	0.73	1.00	0.76	1.00	0.68
	**Adiabatic Anion Radical**							**Adiabatic Cation Radical**					
	**dAdo**			^{OXO}**dAdo**		^{HO}**dAdo**		**dAdo**		^{OXO}**dAdo**		^{HO}**dAdo**	
**dR**	0.01	0.18		0.01	0.11	0.02	0.10	0.00	0.36	0.00	0.25	0.00	0.33
**Ade**	0.99	-1.18		0.99	-1.11	0.98	-1.10	1.00	0.64	1.00	0.75	1.00	0.67
	**Ado**			^{OXO}**Ado**		^{HO}**Ado**		**Ado**		^{OXO}**Ado**		^{HO}**Ado**	
**Ri**	0.02	0.18		0.01	0.08	0.02	0.09	0.00	0.37	0.00	0.23	0.00	0.31
**Ade**	0.98	-1.18		0.99	-1.08	0.98	-1.09	1.00	0.63	1.00	0.77	1.00	0.69

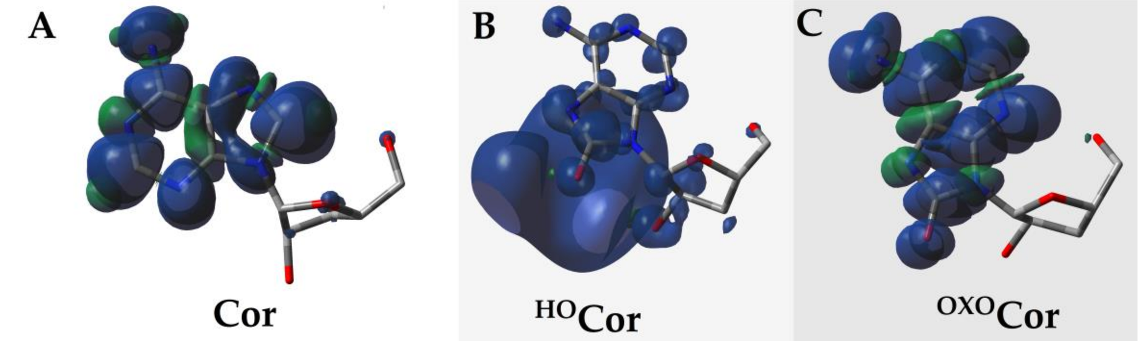

Table S3 The raw data of the charge and spin distribution within dCor, ^{OXO}dCor, ^{HO}dCor and adiabatic radical anion/cation states of canonical nucleosides and their oxidised analogues: Ado, ^{OXO}Ado, ^{HO}Ado, dAdo, ^{OXO}dAdo, ^{HO}dAdo calculated at the M062x/6-31++G** level of theory in the aqueous phase.Hirshfeld charge (**Q**) and spin (**S**) distribution calculated at the M06-2x/6-31++G** level of theory in the aqueous phase . Vertical Cation (^{NE}**VC**) (NE-non-equilibrated), Vertical Cation (^{EQ}**VC**) (EQ-equilibrated), Adiabatic Cation (**AC**) and Vertical Anion (^{NE}**VA**) (NE-non-equilibrated), Vertical Anion (^{EQ}**VA**) (EQ-equilibrated), Adiabatic Anion (**AA**).

7.8-dihydro-8-OXO-3′-deoxyadenosine (^{OXO}dCor)													
**Atom Number**	**Neutral**	^{NE}**VA**		^{EQ}**VN**		**AA**		^{NE}**VC**		**VC**		**AA**	
	**Q**	**S**	**Q**	**S**	**Q**	**S**	**Q**	**S**	**Q**	**S**	**Q**	**S**	**Q**
C1'	0.25	0.00	0.23	0.00	0.23	0.01	0.22	0.00	0.26	0.00	0.27	0.00	0.27
C2'	0.12	0.00	0.11	0.00	0.11	0.00	0.11	0.00	0.13	0.00	0.13	0.00	0.13
O2'	-0.11	0.00	-0.12	0.00	-0.12	0.00	-0.12	0.00	-0.09	0.00	-0.10	0.00	-0.10
C3'	0.03	0.00	0.03	0.00	0.01	0.01	0.02	0.00	0.05	0.00	0.04	0.00	0.05
C4'	0.12	0.00	0.11	0.00	0.11	0.00	0.10	0.00	0.13	0.00	0.12	0.00	0.14
O4'	-0.27	0.00	-0.27	0.00	-0.28	0.01	-0.29	0.00	-0.27	0.00	-0.27	0.00	-0.26
O5'	-0.12	0.01	-0.22	0.00	-0.12	0.01	-0.13	0.00	-0.12	0.00	-0.12	0.00	-0.08
C5'	0.15	0.00	0.11	0.00	0.15	0.00	0.14	0.00	0.17	0.00	0.16	0.00	0.16
N1	-0.39	0.02	-0.45	0.04	-0.47	0.17	-0.49	-0.03	-0.35	-0.03	-0.35	-0.02	-0.36
C2	0.30	0.08	0.19	0.06	0.18	0.02	0.19	0.07	0.41	0.07	0.41	0.09	0.39
N3	-0.36	0.20	-0.45	0.20	-0.48	0.10	-0.43	0.01	-0.31	0.01	-0.31	0.01	-0.33
C4	0.24	0.15	0.17	0.15	0.15	0.36	0.07	0.07	0.29	0.07	0.29	0.08	0.30
**C5**	0.08	0.01	0.05	0.01	0.04	0.06	0.03	0.24	0.18	0.24	0.19	0.24	0.19
C6	0.24	0.31	0.11	0.31	0.10	0.14	0.13	0.07	0.29	0.07	0.30	0.08	0.30
N6	0.08	0.11	-0.08	0.11	-0.09	0.04	-0.06	0.21	0.27	0.22	0.27	0.20	0.28
N7	-0.03	-0.01	-0.07	-0.01	-0.07	0.01	-0.08	0.17	0.08	0.17	0.09	0.18	0.07
C8	0.36	0.06	0.31	0.05	0.31	0.01	0.33	0.00	0.40	0.00	0.40	0.01	0.39
O8	-0.41	0.05	-0.48	0.04	-0.47	0.00	-0.45	0.17	-0.28	0.16	-0.29	0.12	-0.29
N9	-0.27	0.00	-0.28	0.01	-0.28	0.06	-0.30	0.02	-0.24	0.02	-0.24	0.01	-0.25

8-hydroxy-3′-deoxyadenosine (^{HO}dCor)

Atom Number	Neutral	^{NE}VA		^{EQ}VN		AA		^{NE}VC		VC		AA	
	Q	S	Q	S	Q	S	Q	S	Q	S	Q	S	Q
C1'	0.25	0.09	0.18	0.00	0.24	0.00	0.24	0.00	0.27	0.00	0.27	0.00	0.28
C2'	0.13	0.04	0.08	0.00	0.11	0.01	0.11	0.00	0.13	0.00	0.13	0.00	0.13
O2'	-0.11	0.06	-0.17	0.00	-0.12	0.00	-0.12	0.00	-0.09	0.00	-0.10	0.00	-0.09
C3'	0.03	0.00	0.01	0.00	0.01	0.00	0.02	0.00	0.05	0.00	0.04	0.00	0.05
C4'	0.12	0.01	0.10	0.00	0.11	0.00	0.11	0.00	0.13	0.00	0.12	0.00	0.14
O4'	-0.27	0.02	-0.28	0.00	-0.28	0.00	-0.28	0.00	-0.27	0.00	-0.27	0.00	-0.26
O5'	0.15	0.01	0.14	0.00	0.15	0.00	0.15	0.00	0.17	0.00	0.16	0.00	0.16
C5'	-0.12	0.01	-0.13	0.00	-0.12	0.00	-0.13	0.00	-0.12	0.00	-0.12	0.00	-0.08
N1	-0.41	0.00	-0.42	0.00	-0.47	0.08	-0.48	0.00	-0.36	0.00	-0.35	-0.01	-0.36
C2	0.29	0.01	0.25	0.31	0.04	0.46	-0.02	0.07	0.40	0.07	0.40	0.09	0.39
N3	-0.37	0.01	-0.38	0.20	-0.48	0.15	-0.45	0.08	-0.30	0.09	-0.30	0.06	-0.32
C4	0.22	0.04	0.19	0.02	0.19	0.02	0.18	0.08	0.28	0.08	0.28	0.09	0.28
**C5**	0.06	0.05	0.02	0.07	0.00	0.12	-0.03	0.20	0.15	0.20	0.16	0.19	0.16
C6	0.25	0.02	0.22	0.19	0.14	0.06	0.18	0.06	0.29	0.05	0.30	0.07	0.30
N6	0.08	0.02	0.04	0.08	-0.05	0.02	-0.05	0.26	0.30	0.26	0.31	0.21	0.28
N7	-0.42	0.08	-0.48	-0.01	-0.46	0.00	-0.45	0.11	-0.34	0.10	-0.33	0.13	-0.34
C8	0.34	0.14	0.23	0.10	0.28	0.04	0.31	0.09	0.41	0.10	0.41	0.11	0.41
O8	-0.26	0.03	-0.28	0.01	-0.28	0.02	-0.28	-0.01	-0.24	-0.01	-0.24	-0.01	-0.24
N9	0.03	0.38	-0.32	0.03	-0.02	0.01	-0.01	0.07	0.13	0.07	0.12	0.07	0.13

3′-deoxyadenosine (dCor)

Atom Number	Neutral	^{NE}VA		^{EQ}VN		AA		^{NE}VC		VC		AA	
	Q	S	Q	S	Q	S	Q	S	Q	S	Q	S	Q
C1'	0.26	0.01	0.23	0.00	0.23	0.00	0.23	0.00	0.27	0.00	0.27	0.00	0.28
C2'	0.13	0.00	0.12	0.00	0.12	0.00	0.12	0.00	0.13	0.00	0.13	0.00	0.14
O2'	-0.10	0.00	-0.12	0.00	-0.11	0.00	-0.11	0.00	-0.08	0.00	-0.09	0.00	-0.09
C3'	0.06	0.01	0.03	0.01	0.04	0.01	0.04	0.00	0.07	0.00	0.06	0.00	0.07
C4'	0.12	0.00	0.11	0.00	0.12	0.00	0.12	0.00	0.14	0.00	0.13	0.00	0.13
O4'	-0.26	0.00	-0.27	0.00	-0.27	0.00	-0.27	0.00	-0.25	0.00	-0.25	0.00	-0.25
O5'	-0.09	0.00	-0.09	0.00	-0.09	0.00	-0.09	0.00	-0.08	0.00	-0.08	0.00	-0.08
C5'	0.16	0.00	0.14	0.00	0.15	0.00	0.15	0.00	0.18	0.00	0.17	0.00	0.17
N1	-0.41	-0.02	-0.46	-0.02	-0.46	-0.01	-0.45	0.02	-0.35	0.03	-0.35	0.02	-0.35
C2	0.29	0.21	0.11	0.22	0.10	0.17	0.13	0.05	0.40	0.05	0.40	0.07	0.39
N3	-0.40	0.15	-0.50	0.16	-0.51	0.19	-0.50	0.14	-0.30	0.14	-0.30	0.13	-0.31
C4	0.22	0.01	0.19	0.01	0.19	0.02	0.18	0.07	0.27	0.06	0.27	0.07	0.28
**C5**	0.06	0.03	0.03	0.03	0.02	0.03	0.02	0.17	0.14	0.18	0.15	0.17	0.16
C6	0.25	0.22	0.15	0.23	0.14	0.29	0.11	0.05	0.30	0.04	0.31	0.06	0.31
N6	0.09	0.09	-0.05	0.10	-0.05	0.08	-0.10	0.28	0.33	0.29	0.33	0.22	0.31
N7	-0.41	0.03	-0.47	0.03	-0.47	0.02	-0.46	0.08	-0.34	0.06	-0.33	0.09	-0.33
C8	0.29	0.22	0.13	0.20	0.14	0.18	0.16	0.14	0.41	0.14	0.40	0.16	0.40
O8	-0.26	0.03	-0.29	0.02	-0.28	0.01	-0.28	0.00	-0.23	0.00	-0.24	0.01	-0.23
N9	0.26	0.01	0.23	0.00	0.23	0.00	0.23	0.00	0.27	0.00	0.27	0.00	0.28

Atom Number	Adiabatic Anion						Adiabatic Cation					
	Ado		^{OXO}Ado		^{HO}Ado		Ado		^{OXO}Ado		^{HO}Ado	
	S	Q	S	Q	S	Q	S	Q	S	Q	S	Q
C1'	0.00	0.24	0.00	0.24	0.00	0.24	0.00	0.28	0.00	0.27	0.00	0.28
C2'	0.00	0.11	0.01	0.11	0.01	0.11	0.00	0.13	0.00	0.13	0.00	0.13
O2'	0.00	-0.10	0.00	-0.11	0.00	-0.11	0.00	-0.08	0.00	-0.09	0.00	-0.08
C3'	0.01	0.11	0.00	0.09	0.00	0.09	0.00	0.13	0.00	0.11	0.00	0.11
O3'	0.00	-0.10	0.00	-0.11	0.00	-0.11	0.00	-0.08	0.00	-0.09	0.00	-0.09
C4'	0.00	0.12	0.00	0.11	0.00	0.11	0.00	0.13	0.00	0.13	0.00	0.14
O4'	0.00	-0.26	0.00	-0.28	0.00	-0.28	0.00	-0.24	0.00	-0.26	0.00	-0.25
C5'	0.00	0.15	0.00	0.15	0.00	0.15	0.00	0.17	0.00	0.16	0.00	0.17
O5'	0.00	-0.09	0.00	-0.12	0.00	-0.13	0.00	-0.08	0.00	-0.11	0.00	-0.08
N1	-0.01	-0.45	0.08	-0.47	0.08	-0.47	0.02	-0.35	-0.02	-0.35	-0.01	-0.36
C2	0.19	0.12	0.45	0.02	0.46	-0.02	0.07	0.40	0.08	0.40	0.09	0.39
N3	0.18	-0.49	0.09	-0.43	0.15	-0.45	0.13	-0.31	0.02	-0.32	0.06	-0.32
C4	0.01	0.18	0.05	0.19	0.02	0.18	0.07	0.28	0.07	0.30	0.09	0.28
C5	0.03	0.02	0.17	-0.05	0.12	-0.03	0.17	0.16	0.24	0.20	0.19	0.16
C6	0.27	0.12	0.07	0.18	0.06	0.18	0.06	0.31	0.08	0.31	0.07	0.30
N6	0.08	-0.10	0.02	-0.03	0.02	-0.05	0.22	0.31	0.21	0.29	0.21	0.28
N7	0.02	-0.46	0.04	-0.10	0.00	-0.45	0.09	-0.33	0.18	0.08	0.13	-0.34
C8	0.20	0.15	0.00	0.34	0.04	0.31	0.16	0.41	0.01	0.40	0.11	0.41
N9	0.01	-0.28	0.01	-0.28	0.02	-0.28	0.01	-0.23	0.00	-0.25	-0.01	-0.24
O8	--	--	0.00	-0.44	0.01	-0.01	--	--	0.12	-0.29	0.07	0.13

Atom Number	Adiabatic Anion						Adiabatic Cation					
	dAdo		^{OXO}dAdo		^{HO}dAdo		dAdo		^{OXO}dAdo		^{HO}dAdo	
	S	Q	S	Q	S	Q	S	Q	S	Q	S	Q
C1'	0.00	0.23	0.00	0.23	0.00	0.25	0.00	0.27	0.00	0.28	0.00	0.29
C2'	0.01	0.04	0.01	0.04	0.01	0.03	0.00	0.08	0.00	0.06	0.00	0.07
C3'	0.00	0.12	0.00	0.12	0.00	0.09	0.00	0.13	0.00	0.11	0.00	0.11
O3'	0.00	-0.12	0.00	-0.12	0.00	-0.11	0.00	-0.11	0.00	-0.10	0.00	-0.10
C4'	0.00	0.12	0.00	0.12	0.00	0.11	0.00	0.14	0.00	0.13	0.00	0.14
C4'	0.00	-0.28	0.00	-0.28	0.00	-0.28	0.00	-0.26	0.00	-0.26	0.00	-0.26
C5'	0.00	0.15	0.00	0.15	0.00	0.14	0.00	0.17	0.00	0.16	0.00	0.16
O5'	0.00	-0.09	0.00	-0.09	0.00	-0.13	0.00	-0.07	0.00	-0.11	0.00	-0.08
N1	-0.01	-0.45	-0.01	-0.45	0.08	-0.48	0.02	-0.35	-0.02	-0.36	-0.01	-0.36
C2	0.19	0.12	0.19	0.12	0.46	-0.03	0.07	0.39	0.08	0.40	0.09	0.38
N3	0.18	-0.50	0.18	-0.50	0.15	-0.45	0.13	-0.31	0.02	-0.32	0.06	-0.32
C4	0.01	0.18	0.01	0.18	0.03	0.18	0.07	0.28	0.07	0.30	0.09	0.28
C5	0.03	0.02	0.03	0.02	0.13	-0.03	0.17	0.16	0.24	0.19	0.19	0.16
C6	0.27	0.12	0.27	0.12	0.06	0.17	0.06	0.31	0.08	0.31	0.07	0.30
C7	0.08	-0.10	0.08	-0.10	0.02	-0.05	0.23	0.31	0.21	0.29	0.21	0.27
N7	0.02	-0.46	0.02	-0.46	0.00	-0.45	0.08	-0.33	0.18	0.08	0.13	-0.34
C8	0.21	0.16	0.21	0.16	0.04	0.31	0.16	0.41	0.01	0.40	0.11	0.41
N9	0.02	-0.28	0.02	-0.28	0.02	-0.28	0.01	-0.23	0.01	-0.24	-0.01	-0.24
O8	--	--	-0.002	00	00	-0.01	--	--	0.12	-0.29	0.07	0.13

Table S4.

The energies in Hartree calculated at the M06-2x/6-31++G** level of theory in the aqueous phase. Vertical Cation (^{NE}**VC**) (NE-non-equilibrated), Vertical Cation (^{EQ}**VC**) (EQ-equilibrated), Adiabatic Cation (**AC**) and Vertical Anion (^{NE}**VA**) (NE-non-equilibrated), Vertical Anion (^{EQ}**VA**) (EQ-equilibrated), Adiabatic Anion (**AA**). **Ri**: ribose, **2-dR**: 2-deoxyribose, **3-R**: 3-deoxyribose, **Ade**: adenine.

	**AC**	^{EQ}**VC**	^{NE}**VC**	**Neutral**	^{NE}**VA**	^{EQ}**VA**	**AA**
dCor_*N*_Syn	-887.816247	-887.767235	-887.806811	-888.050178	-888.051573	-888.089837	-888.101608
dCor_*N*_anti	-887.823717	-887.774436	-887.813857	-888.054851	-888.054651	-888.093203	-888.105453
dCor_*S*_Syn	-887.814357	-887.765393	-887.804989	-888.048916	-888.050335	-888.089129	-888.100929
dCor_*S*_anti	-887.820323	-887.770945	-887.810586	-888.052561	-888.052956	-888.09157	-888.103629
^{OXO}dCor_*N*_Syn	-963.056615	-963.039573	-963.000918	-963.277226	-963.265524	-963.310353	-963.32841
^{OXO}dCor_*N*_Anti	-963.051814	-963.03926	-963.03926	-963.277173	-963.264185	-963.310589	-963.329378
^{OXO}dCor_*S*_Syn	-963.049794	-963.04638	-963.00797	-963.286365	-963.324694	-963.285894	-963.327684
^{OXO}dCor_*S*_Anti	-963.049521	-963.037465	-962.998844	-963.275283	-963.308959	-963.267835	-963.325465
^{HO}dCor_*N*_Syn	-963.043809	-963.027028	-962.988136	-963.261529	-963.247392	-963.290788	-963.30622
^{HO}dCor_*N*_Anti	-963.036711	-963.023569	-962.984588	-963.260001	-963.2533	-963.291597	-963.305397
^{HO}dCor_*S*_Syn	-963.044219	-963.032285	-963.032073	-963.271113	-963.249599	-963.306541	-963.322077
^{HO}dCor_*S*_Anti	-963.036711	-962.972881	-962.972703	-963.260017	-963.207519	-963.24996	-963.305765

	**Neutral**		**Adiabatic Cation**		**Adiabatic Anion**	
	Type *S*	Type *N*	Type *S*	Type *N*	Type *S*	Type *N*
dAdo Syn	-888.049594	-888.050048	-887.816022	-887.816586	-888.100842	-888.101135
dAdo Anti	-888.054061	-888.052805	-887.822173	-887.822148	-888.10461	-888.103022
^{OXO}dAdo_Syn	-963.276434	-963.277132	-963.051197	-963.052294	-963.327197	-963.328254
^{OXO}dAdo_Anti	-963.276076	-963.276967	-963.051401	-963.052268	-963.327566	-963.328118
^{HO}dAdo_Syn	-963.261418	-963.261555	-963.038134	-963.043708	-963.305679	-963.305875
^{HO}dAdo_Anti	-963.261003	-963.250259	-963.038466	-963.038372	-963.305525	-963.305579
Ado Syn	-963.246196	-963.246637	-963.011681	-963.011978	-963.308324	-963.298278
Ado Anti	-963.249693	-963.250259	-963.016939	-963.018332	-963.301219	-963.301485
^{OXO}Ado Syn	-1038.472326	-1038.473913	-1038.247089	-1038.247688	-1038.529609	-1038.525968
^{OXO}Ado Anti	-1038.457448	-1038.473848	-1038.23396	-1038.247729	-1038.502529	-1038.532169
^{HO}Ado Syn	-1038.460801	-1038.458066	-1038.233577	-1038.238427	-1038.510046	-1038.503417
^{HO}Ado Anti	-1038.457448	-1038.457817	-1038.23396	-1038.23337	-1038.502529	-1038.502017

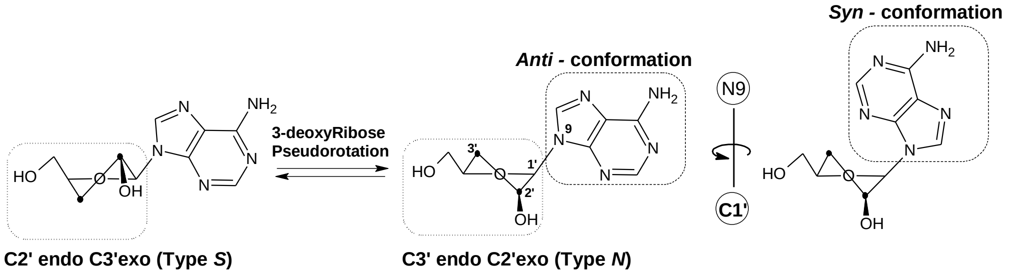

Supplement: Supplementary file 1 [file molecules-30-02289-s001.zip › molecules-3634958-supplementary.pdf]
